# Evaluation of glial tumors: correlation between magnetic resonance imaging and histopathological analysis

**DOI:** 10.1590/0100-3984.2024.0025

**Published:** 2024-09-16

**Authors:** Lillian Gonçalves Campos, Francine Hehn de Oliveira, Ápio Cláudio Martins Antunes, Juliana Ávila Duarte

**Affiliations:** 1 Department of Radiology, Hospital de Clínicas de Porto Alegre (HCPA), Porto Alegre, RS, Brazil; 2 Universidade Federal do Rio Grande do Sul (UFRGS), Porto Alegre, RS, Brazil

**Keywords:** Glioma, Neoplasm grading, Magnetic resonance imaging, Brain neoplasms, Isocitrate dehydrogenase, Glioma, Gradação de tumores, Ressonância magnética, Neoplasias encefálicas, Isocitrato desidrogenase

## Abstract

**Objective:**

To determine the correlation of conventional and diffusion-weighted imaging findings on magnetic resonance imaging (MRI) of the brain, based on Visually AcceSAble Rembrandt Images (VASARI) criteria, with the histopathological grading of gliomas: low-grade or high-grade.

**Materials and Methods:**

Preoperative MRI scans of 178 patients with brain gliomas and pathological confirmation were rated by two neuroradiologists for tumor size, location, and tumor morphology, using a standardized imaging feature set based on the VASARI criteria.

**Results:**

In the univariate analysis, more than half of the MRI characteristics evaluated showed a significant association with the tumor grade. The characteristics most significantly associated with the tumor grade were hemorrhage; restricted diffusion; pial invasion; enhancement; and a non-contrast-enhancing tumor crossing the midline. In a multivariable regression model, the presence of enhancement and hemorrhage maintained a significant association with high tumor grade. The absence of contrast enhancement and restricted diffusion were associated with the presence of an isocitrate dehydrogenase gene mutation.

**Conclusion:**

Our data illustrate that VASARI MRI features, especially intratumoral hemorrhage, contrast enhancement, and multicentricity, correlate strongly with glial tumor grade.

## Abstract (pt)

**Objetivo:**

Determinar a correlação dos achados de imagem convencional e de difusão na ressonância magnética (RM) do encéfalo, com base nos critérios Visually AcceSAble Rembrandt Images (VASARI), com a classificação histopatológica de gliomas: gliomas de baixo grau e gliomas de alto grau.

**Materiais e Métodos:**

Imagens de RM pré-cirúrgicas de 178 pacientes com gliomas cerebrais e confirmação patológica foram avaliadas por dois neurorradiologistas quanto ao tamanho, localização e morfologia do tumor usando um padrão de imagem baseado nos critérios VASARI.

**Resultados:**

Na análise univariada, mais da metade das características avaliadas apresentou associação significativa com o grau do tumor. Hemorragia, restrição à difusão, invasão pial, realce e tumor sem realce cruzando a linha média foram as características com associação mais significativa. No modelo de regressão multivariada, a presença de realce e hemorragia manteve associação significativa com tumores de alto grau. A ausência de realce pelo meio de contraste e a restrição da difusão foram associados à presença da mutação do gene isocitrato desidrogenase.

**Conclusão:**

Nossos dados ilustram que as características de RM do VASARI, especialmente hemorragia intratumoral, presença de realce de contraste e multicentricidade, forneceram uma correlação importante com o grau da neoplasia glial.

## INTRODUCTION

Glioma is one of the most common primary tumors in the central nervous system^(1-3)^. Gliomas are typically classified as low-grade or high-grade based on the histopathological criteria established in 2016 by the World Health Organization (WHO), in which grade 2 indicates a low-grade glioma and grade 3 or 4 indicates a high-grade glioma^(2,4)^. This classification, even today, defines therapeutic strategies and predicts prognosis^(5,6)^. High-grade gliomas are usually highly proliferative, with rapid progression and short patient survival, whereas low-grade gliomas have a better prognosis^(7)^.

The most recent brain tumor classification is the 2021 WHO classification, which incorporates molecular parameters in addition to histology to define many tumor entities^(8,9)^. In this new tumor classification, a mutation in the isocitrate dehydrogenase (IDH) gene is an important molecular marker in glioma diagnosis, with significant implications for tumor behavior and patient prognosis^(9,10)^. Patients who have a glioma with an IDH mutation show better overall survival and response to treatment in comparison with those who have an IDH wild-type glioma^(9,11)^.

The Ki-67 proliferation index is a measure of cell proliferation and can be used as a prognostic marker in gliomas^(12,13)^. A high Ki-67 proliferation index is associated with increased tumor aggressiveness and is commonly higher in high-grade gliomas^(9)^.

Histopathological analysis and, more recently, molecular analysis are the gold-standard techniques for grading intracranial glial tumors^(6)^. However, these procedures require a stereotactic biopsy or resection of the neoplasm, invasive procedures that present risks of complications for the patient and are subject to sampling errors, potentially resulting in inaccurate grading^(9,10)^. In some cases, these tumors are in inaccessible regions, contraindicating sample collection^(1)^. In addition, many of the molecular and immunohistochemical markers are not accessible in lowand middle-income countries. A noninvasive method that allows the definition of the grade and molecular status of brain gliomas is highly desirable and can overcome some of these limitations^(14,15)^. Magnetic resonance imaging (MRI) using an intravenous gadolinium-based contrast agent is a well-established tool for the characterization of intracranial tumors^(16)^. With its superior image resolution and its excellent contrast for soft tissues^(17)^, MRI has been used as a noninvasive diagnostic method for gliomas and usually correlates with histological grade^(15,18)^.

To make the assessment of glioma imaging features more accurate and reproducible, a group of image markers known as Visually AcceSAble Rembrandt Images (VASARI) was defined in 2008^(19,20)^. The VASARI system comprises more than 30 imaging features divided into categories related to location, internal lesion morphology, lesion margin morphology, changes in the proximity of the lesion, and remote changes^(21)^. Previous studies have demonstrated that these features are highly reproducible and clinically significant in gliomas^(19-21)^. Longer progression-free survival, overall survival, and treatment response are examples of clinical markers that could be assessed using VASARI imaging characteristics^(20)^.

Although the VASARI system was created to study gliomas, there have been few studies evaluating the associations between the multiple variables of the system and the histopathological grading of glial tumors^(9,10,19,20,22)^. Many other studies based on the VASARI criteria have used the image bank originally created to develop those criteria^(19,22)^: the Cancer Imaging Archive/Cancer Genome Atlas. Therefore, the analysis of the glial tumor image bank of our institution, in order to correlate the VASARI imaging findings with the WHO glial tumor grade, can reinforce, bring new information, or even indicate a better standardization or search for findings in the MRI evaluation of these tumors.

The aim of this study was to evaluate the potential of VASARI MRI features to provide accurate, valuable information about glioma characteristics, especially glioma grade. We also attempted to determine whether VASARI MRI features correlated with IDH mutation status and the Ki-67 proliferation index.

## MATERIALS AND METHODS

### Patient population

**Figure 1:**
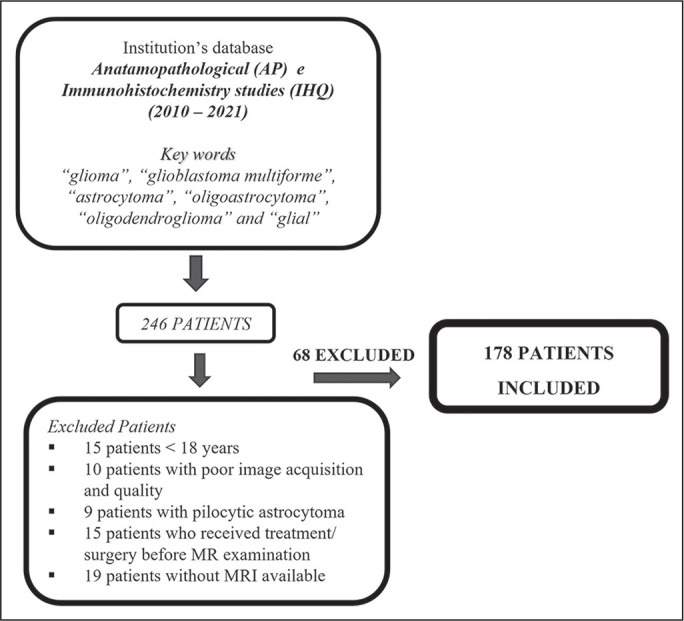
Flow chart of the patient selection process.

In a retrospective review of records added to the glial tumor image bank of our institution between December 2010 and April 2022, we selected all of the pathology and immunohistochemistry studies containing the search terms “glioma”, “glioblastoma multiforme”, “astrocytoma”, “oligodendroglioma”, “oligoastrocytoma” or “glial”. The results of the studies were analyzed, and only those related to patients with a glial tumor for whom MRI scans were available in our image bank were included. The glioma grade was obtained from the final pathology reports. Patients were excluded according to the following criteria: being under 18 years of age; poor image acquisition and quality; having received treatment before MRI examination; and having received a histopathological diagnosis of pilocytic astrocytoma, which, although corresponding to a low-grade lesion (WHO grade 1), presents imaging findings that overlap with those of a high-grade lesion^(21)^. The process of patient inclusion and exclusion is detailed in Figure 1. Immunohistochemistry data for the presence or absence of an IDH1 mutation and to determine the Ki-67 proliferation index were collected when available.

### MRI protocol

All pretreatment MRI scans were acquired either in a 1.5-T scanner (Achieva; Philips Medical Systems, Best, The Netherlands), with an 8-channel sensitivity head-coil, or in a 3.0-T scanner (Ingenia; Philips Medical Systems), with a 16-channel sensitivity head-coil.

The examinations included unenhanced and contrast-enhanced T2-weighted and T1-weighted sequences, together with fluid-attenuated inversion recovery sequences and diffusion-weighted imaging with apparent diffusion coefficient mapping. The MRI examinations were performed in the 1.5-T scanner for 146 patients and in the 3.0-T scanner for 32 patients. Contrast-enhanced sequences were obtained after administration of a gadolinium-based contrast agent (Magnevist; Bayer Healthcare AG, Leverkusen, Germany) at a dose of 0.2 mL/kg of body weight. Detailed parameters of the MRI sequences are presented in Table 1. All scans were deemed to be of diagnostic quality without significant artifacts.

**Table 1:** Parameters used in two MRI scanners.

MRI sequence	Parameter	1.5-T scanner	3.0-T scanner
Axial T2-weighted spin-echo FLAIR	Slice thickness	5.0 mm	4.0 mm
	Pixel/voxel size	0.9 X 0.9 mm	1.1 X 1.1 mm
	Echo time	140 ms	135 ms
	Repetition time	11,000 ms	8,000 ms
	Inversion time	2,200 ms	2,400 ms
	Acquisition matrix	256 X 256	512 X 512
Axial T1-weighted spin-echo	Slice thickness	5.0 mm	4.5 mm
	Pixel/voxel size	0.8 X 0.8 mm	0.9 X 0.9 mm
	Echo time	15 ms	13 ms
	Repetition time	616 ms	660 ms
	Acquisition matrix	256 X 256	512 X 512
Axial T2-weighted spin-echo	Slice thickness	5.0 mm	4.0 mm
	Pixel/voxel size	0.5 X 0.5 mm	0.6 X 0.6 mm
	Echo time	100 ms	92 ms
	Repetition time	5,655ms	3,000 ms
	Acquisition matrix	256 X 256	576 X 576
Axial contrast-enhanced three-dimensional T1-weighted gradient-echo	Slice thickness	2.0 mm	1.0 mm
	Pixel/voxel size	0.7 X 0.7 mm	1.0 X 1.0 mm
	Echo time	3.79 ms	3.5 ms
	Repetition time	25 ms	7.9 ms
	Acquisition matrix	352 X 352	512 X 512
Axial echo-planar DWI with ADC mapping	Slice thickness	3.5 mm	3.5 mm
	Pixel/voxel size	0.7 X 0.7 mm	1.79 X 1.79 mm
	Echo time	77.6 ms	117 ms
	Repetition time	6,629 ms	5,852 ms
	Acquisition matrix	336 X 336	432 X 432

FLAIR, fluid-attenuated inversion recovery; DWI, diffusion-weighted imaging; ADC, apparent diffusion coefficient.

### Dynamic perfusion imaging

Dynamic susceptibility contrast (DSC) MRI perfusion studies were performed in 84 patients. The contrast agent (Magnevist) was administered, at the dosage described above, with a power injector, at a rate of approximately 3.5 mL/s, followed by a saline bolus (10-20 mL at approximately 4 mL/s). Axial gradient-echo echo-planar imaging was performed with the following parameters: repetition time/echo time, 1,800/50 ms; flip angle, 90°; field of view, 24 cm; matrix, 128 × 128; slice thickness, 5 mm; and interslice gap, 1.5 mm. Data were processed on a dedicated workstation (Advantage Windows; GE Healthcare, Chalfont, UK) and transferred to a picture archiving and communication system. Given the retrospective nature of the study, quantitative assessment of relative cerebral blood volume (rCBV) was not possible, because the images were no longer available on the postprocessing workstation. A qualitative visual analysis of the CBV maps was therefore carried out.

### MRI assessment and analysis

The morphologic characteristics and the scoring system were adapted from VASARI feature set for human glioma. This comprehensive feature set contains standardized terminologies of the most common features used to describe primary cerebral neoplasia on standard unenhanced and contrast-enhanced MRI^(22)^. Twenty-five of the characteristics that make up the VASARI feature set were analyzed in addition to the qualitative assessment of the perfusion study based on the CBV color maps. For statistical analysis purposes, some of the characteristics that could be separated into three or more categories were converted to dichotomous variables (Table 2). An open-source picture archiving and communication system workstation (Enterprise Imaging; AGFA HealthCare, Mortsel, Belgium) was used for imaging assessments.

**Table 2:** Adapted VASARI imaging features.

Features and scoring	Definition

Major axis size 1. 0.5-5.0 cm; 2. > 5.0 cm	The diameter of the tumor on its longest axis
Tumor location 1. Lobar^*^; 2. Other locations^†^	Location of the geographic epicenter of the lesion ^*^ Frontal, temporal, parietal, occipital, insular;^†^ basal ganglia, brainstem, cerebellum, thalamus
Side of tumor epicenter 1. Right or left; 2. Bilateral/central	Laterality of the lesion epicenter
Eloquent brain area^*^ involvement 1. Yes; 2. No	Does the geographic epicenter or the enhancing component involve an eloquent area of the brain? ^*^ Speech motor, speech receptive, motor, vision
Enhancement quality 1. None; 2. Minimal^*^; 3. Marked^*^	Qualitative degree of contrast enhancement ^*^ Grouped so that the presence of enhancement is in a single category
Proportion CET 1. 0%^*^; 2. < 5%^*^; 3. 6-33%^*^; 4. 34-67%^†^; 5. 68-95%^†^; 6. > 95%^†^	Assuming that the entire abnormality may be comprised of an enhancing component, a non-enhancing compo-nent, a necrotic component, and an edema component, what proportion of the tumor is enhancing? ^*^ 0-33% (grouped categories);^†^ > 34% (grouped categories)
Proportion nCET 1. 0%^*^; 2. < 5%^*^; 3. 6-33%^*^; 4. 34-67%^*^; 5. 68-95%^†^; 6. > 95%^†^	An nCET is defined as regions of T2WI hyperintensity (less than the intensity of the cerebrospinal fluid, with cor-responding T1WI hypointensity. In view of that, what proportion of the tumor is non-enhancing? ^*^ 0-33% (grouped categories);^†^ > 34% (grouped categories)
Proportion necrosis 1. 0%^*^; 2. < 5%^*^; 3. 6-33%^†^; 4. 34-67%^†^; 5. 68- 95%^†^; 6. > 95%^†^	Defined as a region within the tumor that does not enhance or shows markedly diminished enhancement, with hyperintensity on T2WI, hypointensity on T1WI, and an irregular border ^*^ < 5% (grouped categories); ^†^> 5% (grouped categories)
Cysts 1. Yes; 2. No	Well-defined, rounded, often eccentric regions of very high and low signal intensity on T2WI and T1WI, respec-tively, essentially matching that of the cerebrospinal fluid, with very thin, regular, smooth, non-enhancing or regularly enhancing walls, possibly with thin, regular, internal septations
Multifocal or multicentric 1. No; 2. Multifocal^*^; 3. Multicentric^*^; 4. Gliomatosis^*^	Multifocal is defined as having at least one region of tumor, either enhancing or non-enhancing, which is not contiguous with the dominant lesion. Multicentric is defined as widely separated lesions in different lobes or dif-ferent hemispheres. Gliomatosis refers to the generalized neoplastic transformation of the white matter of most of a hemisphere ^*^ Grouped into one category
Tl/FLAIR ratio 1. Expansive; 2.Mixed^*^; 3. Infiltrative^*^	Expansive = size of the abnormality on an unenhanced T1WI approximates that seen on a FLAIR sequence. Mixed = size of the abnormality on an unenhanced T1WI moderately less than that seen on a FLAIR sequence. Infiltrative = size of the abnormality on an unenhanced T1WI much smaller than that seen on a FLAIR sequence ^*^ Grouped into one category
Thickness of enhancing margin 1. Not applicable; 2. None; 3. Thin; 4. Thick	The scoring is not applicable if there is no contrast enhancement. If most of the enhancing rim is thin, regular, and homogenous, the grade is thin. If most of the rim demonstrates nodular or thick enhancement, the grade is thick. If there is only solid enhancement and no rim, the grade is none
Definition of enhancing margin 1. Not applicable; 2. Well-defined; 3. Poorly defined	The score is not applicable if there is no contrast enhancement. Assess if most of the outside margin of the en-hancement is well defined or poorly defined
Definition of the non-enhancing margin 1. Smooth; 2. Irregular; 3. Not applicable	If most of the outside non-enhancing margin of the tumor is well defined and smooth (geographic), versus poorly defined and irregular
Proportion of edema 1. 0%^*^; 2. < 5%^*^; 3. 6-33%^t^; ; 4. 34-67%^†^; 5. 68-95%^†^; 6. > 95%^†^	What proportion of the abnormality is vasogenic edema? Edema should be greater in signal than nCET ^*^ < 5% (grouped categories);^†^ > 5% (grouped categories)
Edema crossing the midline 1. Yes; 2. No	Edema spans white matter commissures extending into the contralateral hemisphere
Hemorrhage 1. No; 2. Yes	Intrinsic hemorrhage in the tumor matrix. Any intrinsic foci of low signal intensity on T2WI and SWI or high signal intensity on T1WI
Diffusion 1. Facilitated^*^; 2. Restricted; 3. Neither/equivocal^*^	Predominantly facilitated or restricted diffusion in the enhancing or non-enhancing portion of tumor. (Based on the ADC map). The proportion of tissue is not relevant ^*^ Grouped into one category
Pial invasion 1. Yes; 2. No	Enhancement of the overlying pia in continuity with CET or nCET
Ependymal invasion 1. Yes; 2. No	Invasion of any adjacent ependymal surface in continuity with CET or nCET matrix
Cortical involvement 1. Yes; 2. No	CET or nCET extending to the cortical mantle, or cortex is no longer distinguishable relative to the subjacent tumor
Deep white matter invasion 1. Yes; 2. No	CET or nCET extending into the internal capsule or brainstem
nCET crossing the midline 1. Yes; 2. No	nCET crosses into the contralateral hemisphere through white matter commissures
CET crossing the midline 1. Yes; 2. No	Enhancing tissue crosses into the contralateral hemisphere through white matter commissures
Satellites 1. Yes; 2. No	A satellite lesion is an area of enhancement within the region of signal abnormality surrounding the dominant lesion but not contiguous in any part with the major tumor mass

CET, contrast-enhancing tumor; nCET, non-contrast-enhancing tumor; T2WI, T2-weighted imaging; T1WI, T1-weighted imaging; FLAIR, fluid-attenuated inversion recovery; SWI, susceptibility-weighted imaging; ADC, apparent diffusion coefficient.

The imaging features plus a single measurement of lesion size were evaluated by two neuroradiologists, each with more than 10 years of experience. Initially, each neuroradiologist made the assessments independently. Interrater agreement between the two independent values was calculated. To obtain a single result for each characteristic evaluated, disagreements were resolved by consensus. The neuroradiologists were blinded to the pathology results corresponding to the images analyzed.

### Histopathology and molecular analysis

The results of the pathology studies were obtained from patient medical records. All tissue samples were biopsy specimens or fresh surgical tissues, preserved in formalin-fixed paraffin-embedded blocks. All samples were evaluated by a pathologist, who categorized them according to the WHO classification of tumors of the central nervous system. The brain glioma grade was determined on the basis of morphological criteria. The IDH status was determined by mutation-specific immunohistochemistry for the most common IDH1 mutation (R132H). The monoclonal antibody MIB-1 was used in order to identify Ki-67 in paraffin-embedded sections. The Ki-67 index is expressed as the percentage of positively stained nuclei.

### Statistical analysis

The statistical analysis was conducted with the Predictive Analytics Software package, version 18.0 (SPSS Inc., Chicago, IL, USA). Because all continuous variables in this study were normally distributed, they are expressed as means ± standard deviations. Interrater agreement for the VASARI features was assessed by calculating Cohen’s kappa (κ). Univariate analysis was employed to identify covariates that might affect the grading of a glioma. The chi-square test and Fisher’s exact test were performed on the univariate analysis to compare the predictive factors. A binary logistic regression followed by a stepwise binary logistic regression analysis was applied to identify the significant independent factors for predicting the glioma grade. All MRI characteristics that showed a significant association with tumor grade in the univariate analysis were considered in the multivariate analysis. Linear regression was also performed to determine whether Ki-67 values were associated with any VASARI feature. The significance was set at a value of 0.05. Receiver operating characteristic curve analysis was used in order to assess the performance of the VASARI features most strongly associated with tumor grade.

## RESULTS

### Interrater agreement

Interrater agreement for the MRI scoring ranged from moderate to almost perfect. The level of that agreement was highest (almost perfect) for the presence of cysts (κ = 1.0) and contrast enhancement (κ = 0.82). The level of agreement was substantial for hemorrhage (κ = 0.7), necrosis (κ = 0.8), and restricted diffusion (κ = 0.66), whereas it was moderate for other features such as perfusion imaging (κ = 0.6) and involvement of eloquent areas of the brain (κ = 0.6).

### Patient and tumor characteristics

Our study sample comprised 178 patients. The characteristics of the patients and their tumors are summarized in Table 3. Of the 178 patients included in the analysis, 76 (42.7%) were female. The mean age was 53.7 ± 14.6 years. Of the 178 corresponding tumors, 140 (78.6%) were found to be high-grade gliomas, the remaining 38 (21.3%) being categorized as low-grade gliomas. High-grade tumors were significantly more common in males than in females (*p* < 0.05).

**Table 3:** Patient and tumor characteristics.

Variable	(N = 178)
Histopathological grade, n (%)	
High	140 (78.7)
Low	38 (2.3)
Sex, n (%)	
Female (all)	76 (42.7)
Female with a high-grade tumor	53 (69.7)
Female with a low-grade tumor	23 (30.3)
Age (years), mean ± standard deviation	
All patients	53.7 ± 14.6
Patients with a high-grade tumor	55.8 ± 13.7
Patients with a low-grade tumor	46.1 ± 15.7
Histological type, n (%)	
Glioblastoma	111 (62.4)
WHO grade 2 astrocytoma	19 (10.7)
WHO grade 3 astrocytoma	16 (9.0)
Other	32 (17.9)
Immunohistochemistry, n (%)	
IDH mutation	47 (26.0)
Ki-67	76 (42.7)

The most common histological type was glioblastoma (identified in 62.4%), followed by WHO grade 2 and grade 3 astrocytomas, which collectively accounted for almost 20% of the tumors evaluated. The Ki-67 assay was performed in 76 (42.7%) of the tumors. Immunohistochemical analysis to detect an IDH1 mutation was performed in 47 (26.4%).

The MRI features evaluated, based on VASARI criteria, are described in Table 4. Contrast enhancement and necrosis were found in more than 85% of the patients. Satellite lesions and multicentricity were less prevalent findings, occurring in less than 25% of the tumors. We performed DSC MRI perfusion in 47% of the tumors, of which 75% showed increased CBV.

**Table 4:** MRI features in patients with glioma (N = 178).

Feature	n (%)
Tumor location	
Lobar	119 (66.9)
Other (deep)	59 (33.1)
Necrosis	
Yes	161 (90.4)
No	17 (9.6)
Deep white matter invasion	
Yes	76 (57.3)
No	102 (42.7)
Enhancement	
Yes	153 (86.0)
No	23 (13.9)
Laterality	
Right/left	157 (88.2)
Central/bilateral	21 (11.8)
CET crossing the midline	
Yes	36 (20.2)
No	140 (78.7)
Multicentric/multifocal	
Yes	41 (23.0)
No	137 (77.0)
Cysts	
Yes	18 (10.1)
No	160 (89.9)
nCET crossing the midline	
Yes	84 (47.2)
No	94 (52.8)
Tl/FLAIR	
Expansive	53 (30.0)
Infiltrative	125 (70.0)
Edema crossing the midline	
Yes	74 (41.6)
No	104 (58.4)
Satellite lesions	
Yes	36 (20.2)
No	140 (78.7)
Edema proportion	
<5%	149 (83.7)
>5%	29 (16.3)
Hemorrhage	
Yes	117 (65.7)
No	58 (41.6)
Cortical involvement	
Yes	136 (76.4)
No	42 (23.6)
Restricted diffusion	
Yes	47 (26.4)
No	126 (70.8)
Pial invasion	
Yes	61 (34.3)
No	115 (64.6)
Eloquent brain area	
Yes	59 (33.1)
No	119 (66.9)
Diameter	
0.5-5.0 cm	60 (33.7)
> 5.0 cm	118 (66.3)
Ependymal invasion	
Yes	123 (69.1)
No	55 (30.9)
Enhancement proportion	
0-33%	131 (73.6)
34-100%	45 (25.3)
Perfusion^*^	
Increased	63 (75.0)
Decreased	21 (25.0)

CET, contrast-enhancing tumor; nCET, non-contrast-enhancing tumor; T1/FLAIR, T1-weighted/fluid-attenuated inversion recovery.

* Data available for only 84 (47.2%) of the 178 patients.

### Predictors of tumor grade

In the univariate analysis, more than half of the characteristics evaluated showed a significant association with the tumor grade (Table 5). Hemorrhage, restricted diffusion, pial invasion, enhancement, and a non-contrast-enhancing tumor crossing the midline were the characteristics with the most significant association (*p* < 0.01 for all). For predicting high-grade tumors, hemorrhage within the tumor and contrast enhancement showed odds ratios (ORs) of 7.1 (*p* < 0.01) and 10.6 (*p* < 0.01), respectively. Other good predictors of high-grade tumors in the univariate analysis were restricted diffusion (OR = 2.7; *p* < 0.01) and the presence of pial invasion (OR = 5.9; *p* < 0.01). Non-contrast-enhancing tumor proportion, enhancement quality, and enhancing tumor proportion were also associated with the tumor grade (*p* < 0.01). However, the presence of cysts was associated with low-grade lesions (OR = 3.5; *p* < 0.01). In our analysis, tumor grade showed no association with MRI-confirmed necrosis (*p* > 0.05), lesion size (*p* > 0.05), or ependymal invasion (*p* > 0.05). Nevertheless, the proportions of edema and necrosis within the tumor showed an association with tumor grade (*p* < 0.01 for both).

**Table 5:** Univariate analysis of the associations between MRI features and high-grade glial tumors.

Variable	OR	95% Cl	P-value
Deep tumor	1.5	0.68-3.4	0.3
Central/bilateral	2.8	0.63-12.7	0.16
Eloquent area	0.9	0.4-2.0	0.8
No cysts	3.5	1.3-9.5	**< 0.01**
Infiltrative	0.5	0.2-1.1	0.08
Edema crossing the midline	2.4	1.07-5.2	**< 0.05**
Hemorrhage	7.1	3.2-15.7	**< 0.01**
Restricted diffusion	2.7	1.0-7.5	**< 0.01**
Pial invasion	5.9	2.0-17.8	**< 0.01**
Ependymal invasion	1.6	0.78-3.4	0.2
Cortical involvement	0.5	0.21-1.4	0.2
Deep white matter invasion	1.3	0.6-2.8	0.4
nCET crossing the midline	3.2	1.4-6.9	**< 0.01**
CET crossing the midline	3.7	1.06-12.7	**< 0.05**
Satellite lesions	3.6	1.06-12.7	**< 0.05**
Enhancement	10.6	4.0-27.8	**< 0.01**
Multifocal/multicentric	3.0	1.0-9.2	**< 0.05**
Necrosis	1.2	0.3-3.7	0.8
Necrosis proportion > 5%	7.2	3.2-16.0	**< 0.01**
Edema proportion > 5%	9.2	1.2-70.3	**< 0.01**
Enhancement proportion > 34%	2.9	1.0-7.4	**< 0.05**
Non-enhancement proportion < 67%	3.9	1.4-10.5	**< 0.01**
Diameter > 5.0 cm	0.9	0.4-1.9	0.75

nCET, non-contrast-enhancing tumor; CET, contrast-enhancing tumor; CI, confidence interval.

**Figure 2:**
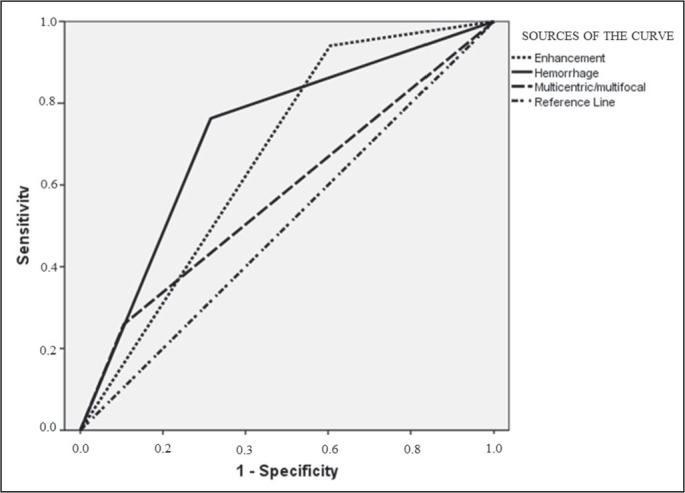
Receiver operating characteristic curve demonstrating individual performance of enhancement, hemorrhage, and multicentricity in tumor grade prediction. Enhancement: area under the curve (AUC), 0.67; sensitivity, 94.0%; specificity, 60.0%. Hemorrhage: AUC, 0.73; sensitivity, 76.0%; specificity, 32.0%; Multicentric: AUC, 0.57; sensitivity, 26.0%; specificity, 10.0%.

**Figure 3:**
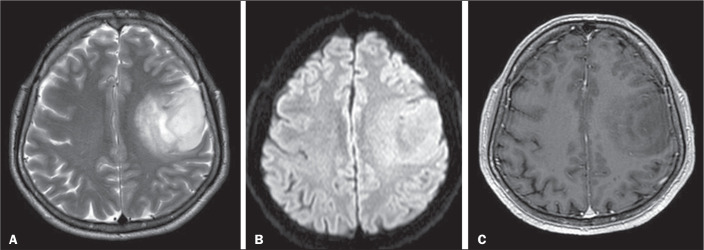
MRI scans of a 37-year-old male patient with a WHO grade 2, IDH-mutant type oligodendroglioma in the left frontoparietal region. A-C: Axial T2-weighted, T2-weighted fluid-attenuated inversion recovery, and contrast-enhanced T1-weighted scans, respectively, showing cortical involvement, no cystic lesion, smooth margins, mild edema around the tumor, and no contrast enhancement.

**Figure 4:**
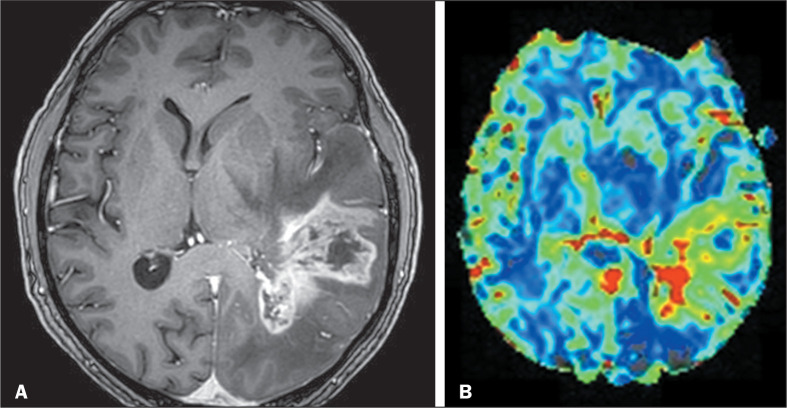
MRI scans of a 63-year-old male patient with a WHO grade 4, IDH-wild type glioblastoma in the left temporoparietal region. A: Axial contrast-enhanced T1-weighted scan showing a large tumor with poorly defined borders, an infiltrative aspect, pronounced contrast enhancement, and subependymal invasion. B: DSC MRI perfusion with high CBV inside the tumor, indicating neoangiogenesis.

In a multivariable regression model (Table 6), enhancement and hemorrhage maintained significant associations with high-grade tumors (OR = 3.1; *p* < 0.05 for both). In stepwise logistic regression (Table 7), intratumoral hemorrhage, contrast enhancement, and multicentricity maintained strong associations with high-grade glial tumors. According to our receiver operating characteristic curve analysis, depicted in Figure 2, the characteristic that performed best for prediction of the tumor grade was hemorrhage (area under the curve: 0.73; sensitivity: 76%; specificity: 32%). Examples of typical imaging findings, illustrating these characteristics, are presented for high-grade tumors in Figure 3 and for low-grade tumors in Figure 4. In the subgroup of 84 patients for whom DSC MRI perfusion sequences were available, increased perfusion showed an association with high-grade tumors (OR = 18.8; *p* < 0.01).

**Table 6:** Multivariate analysis of the associations between MRI features and high-grade glial tumors.

Variable	OR	95% Cl	P-value
No cysts	0.3	0.1-1.1	0.07
Edema crossing the midline	1.07	0.3-3.6	0.9
Hemorrhage	3.1	1.2-8.6	**< 0.05**
Pial invasion	2.6	0.7-9.1	0.14
nCET crossing the midline	1.3	0.3-5.4	0.7
CET crossing the midline	1.3	0.3-7.0	0.7
Satellite lesions	1.5	0.4-6.7	0.5
Enhancement	3.1	1.0-10.4	**< 0.05**
Multifocal/multicentric	2.3	0.5-9.7	0.2
Edema proportion > 5%	3.9	0.45-32.0	0.2
Non-enhancement proportion < 67%	2.3	0.7-7.3	0.18
Necrosis proportion > 5%	2.0	0.6-8.5	0.2
Restricted diffusion	1.4	0.4-4.8	0.6
Enhancement proportion > 34%	0.4	0.1-2.3	0.3

nCET, non-contrast-enhancing tumor; CET, contrast-enhancing tumor; CI, confidence interval.

**Table 7:** Stepwise multivariate analysis of the associations between MRI features and high-grade glial tumors.

Variable	OR	95% Cl	P-value
Hemorrhage	3.6	1.3-9.1	**< 0.01**
Pial invasion	2.9	0.8-9.5	0.08
Enhancement	4.3	1.4-12.8	**< 0.01**
Multifocal/multicentric	3.5	1.0-11.9	**< 0.05**

CI, confidence interval.

### Predictors of IDH-mutant glioma

The absence of contrast enhancement was associated with the presence of an IDH mutation (OR = 17.0; *p* < 0.01) in the subgroup of patients for whom those data were available. The absence of restricted diffusion was another finding that showed a significant correlation with an IDH mutation (OR = 3.9; *p* < 0.04). None of the other imaging findings studied for correlation with the IDH mutation in gliomas showed a statistically significant association with tumor grade (Table 8).

**Table 8:** Univariate analysis of the associations between MRI features and IDH-mutant gliomas.

Variable	OR	P-value
No hemorrhage	1.2	0.75
Decreased perfusion^*^	4.0	0.6
No enhancement	17.0	**< 0.01**
Lobar location	1.7	0.5
No multifocality	4.4	0.15
No pial invasion	2.0	0.4
No restricted diffusion	3.9	**<0.05**

* Data available for only 21 (11.8%) of the 178 patients.

### Predictors of the Ki-67 value

Multifocal lesions, multicentric lesions, and pial invasion were the only imaging features that showed a significant association with Ki-67 values in the linear regression analysis (*p* < 0.05). All of the imaging findings analyzed are shown in Table 9.

**Table 9:** Linear regression of the associations between MRI features and Ki-67 values.

Variable	P-value
CET crossing the midline	0.5
Edema crossing the midline	0.5
Hemorrhage	0.19
Pial invasion	**< 0.05**
nCET crossing the midline	0.8
Satellite lesions	0.7
Enhancement	0.4
MuIticentric/muItifocal	**< 0.05**

CET, contrast-enhancing tumor; nCET, non-contrast-enhancing tumor

## DISCUSSION

In this study, contrast enhancement and hemorrhage were the imaging findings most strongly associated with high-grade glial neoplasms, an association maintained even in the multivariate analysis and stepwise multivariate analysis. Conventional MRI readily provides evidence of contrast enhancement, indicating a breakdown of the blood-brain barrier, which is often associated with high-grade tumors^(16)^. Therefore, MRI enhancement features are of high value for the grading of a glioma. In the present study, most high-grade tumors showed some enhancement, whereas most low-grade tumors showed no enhancement, which is consistent with reports in the literature^(2,23,24)^. Another important finding is that the association with other tumor characteristics in the imaging study, such as hemorrhage and necrosis, can bring greater specificity to the relationship between contrast enhancement and tumor grade^(25,26)^. However, despite this association between contrast enhancement and more aggressive lesions, about a third of low-grade gliomas show some kind of contrast enhancement on baseline neuroimaging^(27-29)^. In our multivariate analysis, the proportions of enhancement and non-enhancement using the VASARI criteria (enhancement proportion > 34% and non-enhancement proportion < 67%) did not show any significant association with lowor high-grade gliomas. Other studies have shown an association between higher proportional enhancement (34-67%) and high-grade tumors. Setyawan et al.^(9)^ stated that this is a potential tool to distinguish between lowand high-grade gliomas, an opinion shared by other authors^(9,10,19)^. It is possible that we obtained different results because of the way the subgroups were divided in our study, in which we chose to dichotomize some of the VASARI characteristics. Therefore, we did not compare the degrees of enhancement (absent, mild, or marked), allocating any intensity of enhancement to the same group. Some studies evaluating the degree of tumor enhancement have found a correlation between mild enhancement and low-grade tumors^(9,10,19)^.

In our subgroup of patients in whom the immunohistochemical study revealed an IDH1 mutation, the absence of contrast enhancement was strongly associated with the IDH1 mutation. Similarly, IDH wild-type tumors in our sample were related to the presence of contrast enhancement, which is in line with other data in the literature^(2,11,23,30)^. Other studies, such as that conducted by Delfanti et al.^(31)^, did not show significant differences between the mutated and IDH wild-type groups, probably because the authors analyzed grade II and grade III tumors exclusively.

Our study, like two others^(2,19)^, showed that intratumoral hemorrhages tend to be much more common in high-grade neoplasms. The high vascular density and the major invasive component of high-grade tumors must be the determinants of this finding^(2,32)^. However, only in our study did hemorrhage remain a predictor of high-grade glial lesions in the multivariate analysis. We did not find a decisive explanation for that difference, although improving the criteria for defining intratumoral hemorrhage could bring greater clarity to these data. On MRI, it is not always possible to distinguish hemorrhage from calcification or hemorrhage from intratumoral vessels. One limitation of our study was that we did not look for calcifications in the lesions detected by computed tomography (CT), which is the gold standard for that alteration, because CT scans were not always available. At some point, integrating MRI findings with those of CT might bring greater clarity to this association^(33)^.

The presence of necrosis was associated with high-grade glial tumors only when the proportion criterion (cutoff of 5%) was used. The simple presence or absence of this finding (regardless of proportion) proved to be insufficient to demonstrate statistical significance, contrary to what has been found in many other studies, which showed a strong association between the presence of necrosis and high-grade tumors^(2,18,19,23,34)^. This can be attributed to differences in measurement and grading systems; that is, quantitative and qualitative assessment^(22)^. Likewise, discrepancies between tumor sampling by biopsy and true tumor grading could have generated this type of disagreement^(35)^. For example, it is possible that areas without histopathological findings, such as those with necrosis and vascular proliferation, were not sampled in a given high-grade tumor, which might thus have been categorized as low-grade.

In our stepwise multivariate analysis, multicentricity and multifocality were associated with high-grade tumors, as demonstrated elsewhere^(19)^. However, as in previous studies^(23)^, this association did not occur with the IDH wild-type tumors in our sample, probably because of the small number of patients evaluated.

Diffusion-weighted imaging is a noninvasive modality that can provide direct insight into the microscopic physical properties of tissues through observation of the Brownian movement of water, which reflects the cellularity within lesions^(35)^. In our univariate analysis, restricted diffusion was significantly associated with high-grade gliomas and IDH wild-type tumors, which is in agreement with most of the data in the literature^(32,36,37)^. However, this association did not remain significant in our multivariate analysis, perhaps reflecting the subjective nature of this VASARI criterion, as evidenced by the fact that the level of interrater agreement was only substantial. The measurements of apparent diffusion coefficient values are more reliable in these situations^(34,37,38)^, and we did not perform these measurements in our study, because we relied on VASARI criteria, which are generally qualitative measurements^(22)^.

Advanced MRI techniques such as perfusion MRI have increasingly been found to be useful in studying brain tumors. It has been shown that CBV maps and measurements correlate reliably with tumor grade and histologic findings of increased tumor vascularity^(16,38,39)^. The increase in CBV in the perfusion study by qualitative assessment was associated with high-grade tumors in our sample. Nonetheless, we observed only moderate interrater agreement for the perfusion evaluation, indicating the low precision of this information. The most likely reason for that is that in many cases the postprocessing could not be redone, and the quality of the reconstructed maps was sometimes dubious.

We did not find an association between ependymal invasion and tumor grade using the VASARI criteria, and this finding is also not well defined in the literature, especially when considering multivariate analyses^(19,38,40)^. This is probably because the criterion is quite broad and sensitive, and we interpreted ependymal invasion as any signal alteration, with or without enhancement, maintaining contact with and altering the signal of the subependymal surface. Perhaps, if subependymal enhancement alone had been included in this category, we would have found a significant correlation between this finding and high-grade tumors^(38)^. A correlation of subependymal invasion by VASARI criteria has been demonstrated only in survival studies, in which it was correlated with a worse prognosis^(22)^.

Signs of cortical invasion by imaging showed no relationship with tumor grade, which was an expected finding given the known distribution of these tumors in the central nervous system. Many oligodendrogliomas or more precisely IDH-mutant 1p/19q codeletion tumors characteristically compromise the brain cortex and do not, therefore, present the behavior of high-grade lesions. Many studies have demonstrated an association between IDH-mutant 1p/19q codeletion tumors and signs of cortical invasion on MRI, regardless of tumor grade^(33)^.

We found no statistically significant association between tumors at deep locations or involvement of deep structures, such as the internal capsule and thalamus, with high-grade lesions, as demonstrated elsewhere^(23)^. That could be explained by the fact that the proportion of lobar tumors was higher in our high-grade lesion group.

The Ki-67 protein is a cellular marker associated with cell proliferation and can objectively reflect tumor aggression. Some studies have shown that the Ki-67 proliferation index is significantly higher in high-grade gliomas than in low-grade gliomas^(12)^. However, few studies have shown a correlation between higher Ki-67 values and morphological imaging findings in gliomas. We demonstrated that Ki-67 values were at least 11% higher in patients with signs of pial invasion or multifocality/multicentricity on MRI. Such imaging findings are often related to high-grade glial lesions, which somewhat supports this positive association with Ki-67 values. Certainly, more studies specifically evaluating the Ki-67 index and imaging findings in glioma are needed for any conclusions to be drawn.

Our study has several limitations. First, it was a retrospective study in which all enrolled patients were treated at the same hospital. More prospective studies are needed. Second, although we reviewed all conventional MRI sequences and diffusion-weighted imaging sequences, some advanced MRI sequences were not available for all patients or simply were not performed. These techniques, especially DSC MRI perfusion, have been shown to correlate well with glial neoplasm grade^(34,38)^. The perfusion study data were available for less than half of the patients and, when present, the images did not always allow a reliable evaluation. We also did not use spectroscopy data for any of the examinations in our sample, because most of the information was incomplete. The small sample size also limits the ability to identify statistically significant predictors of tumor type. Unfortunately, because most patients were diagnosed before techniques for the detection of the IDH mutation became available, we were not able to make a more accurate correlation of this marker with imaging findings. In addition, we did not correlate our imaging findings with patient survival, as has been done in other studies^(18,26,40)^. Given the new perspectives of molecular and genetic analysis, that could have provided us with more practical data on imaging markers indicative of worse survival or higher mortality.

## CONCLUSIONS

We have demonstrated the potential of conventional MRI features for predicting glioma grade and identified imaging features that are related to highand low-grade gliomas. This insight might be helpful when a brain biopsy cannot be performed or when the pathology findings are inconclusive. Our findings also indicate a possible correlation between MRI findings and the IDH1 mutation. Some imaging markers might also be associated with the Ki-67 index.
